# Causal Inference: Onward and Upward!

**DOI:** 10.1177/00220345221084283

**Published:** 2022-03-20

**Authors:** S. Listl, Y. Matsuyama, H. Jürges

**Affiliations:** 1 Radboud University Medical Center - Radboud Institute for Health Sciences (RIHS), Department of Dentistry - Quality and Safety of Oral Healthcare, Nijmegen, the Netherlands; 2 Department of Global Health Promotion, Tokyo Medical and Dental University (TMDU), Bunkyo-ku, Japan; 3 Schumpeter School of Business and Economics, University of Wuppertal, Wuppertal, Germany

**Keywords:** epidemiology, health services research, oral-systemic disease(s), public health, publishing, social determinants

## Abstract

When announcing the Sveriges Riksbank Prize in Economic Sciences in Memory of Alfred Nobel 2021, the Royal Swedish Academy emphasized how conclusions about cause and effect can be drawn from natural experiments. But what can dental research learn from this? The economist’s toolbox provides a number of methods for causal inference from observational data such as instrumental variables, regression discontinuity designs, or difference-in-differences analyses. Although the relevance of improving causal inference in dental research has repeatedly been highlighted in recent years, dental research still seems to reveal major room for improvement in the application of such methods. First, there seems to be an absence of causal literature on key essential research questions for oral health. Second, the diversity and diffusion of causal inferential methods in the dental literature seem very limited so far. Third, while dental research has widely been promoting the use of directed acyclic graphs (DAGs) to help conceptualize causal thinking, comparably little attention seems to have been paid to choosing and applying appropriate data-analytic approaches for causal inference. Fourth, similar to other fields of medicine, confusion seems to persist within the dental research community as to the use of causal language. If dental research is to secure a robust evidence base for promoting effective oral health interventions, we argue that dental research needs to move beyond its current methodological echo chamber and embrace a radically different approach to causal inference. We call for editors, reviewers, and authors to embrace a much more critically reflective approach to causal inference.

When announcing the Sveriges Riksbank Prize in Economic Sciences in Memory of Alfred Nobel 2021, the Royal Swedish Academy emphasized that “this year’s Laureates—David Card, Joshua Angrist and Guido Imbens—have . . . shown what conclusions about cause and effect can be drawn from natural experiments. Their approach has spread to other fields and revolutionised empirical research” (Royal Swedish Academy of Sciences 2021). But what can dental research learn from this?

Causal inference refers to the process of drawing a conclusion that a specific treatment (i.e., intervention) was a “cause” of some observed “effect” or outcome (Gelman and Imbens 2013). Causal inference is highly relevant for dental research as it concerns the deciphering of mechanisms through which oral health can be influenced and mechanisms through which oral health affects people’s health and well-being. This is essential for the development, implementation, and evaluation of oral health interventions and programs.

The economist’s toolbox provides a number of methods for causal inference from observational data such as instrumental variables (IVs), regression discontinuity designs (RDDs), or difference-in-differences (DiD) analyses (Angrist and Pischke 2008; Cunningham 2021; Huntington-Klein 2021). Natural experiments can be particularly useful in the absence of randomized trials (Listl et al. 2016; Hernán and Robins 2020). Although the relevance of improving causal inference in dental research has repeatedly been highlighted in recent years, dental research still seems to reveal major room for improvement in the application of such methods (Listl et al. 2016; Raittio and Farmer 2021).

First, there seems to be an absence of causal literature on key research questions for oral health. For example, methodological gaps in studying the oral–systemic disease connection have recently been highlighted (Seitz et al. 2019; Raittio and Farmer 2021). Another example can be found in research on oral health inequalities. A large amount of evidence stems from descriptive analyses of social inequalities in oral health, and this is, of course, highly relevant to inform health policy. However, evidence on the causal mechanisms of such inequalities is also very much needed. Yet studies on causal effects of socioeconomic status on oral health remain sparse (Matsuyama et al. 2019).

Second, the diversity and diffusion of methods for causal inference from observational data seem relatively limited within the existing dental literature. It is also worth mentioning that a considerable amount of causal analyses on oral health have previously been published outside the dental literature (e.g., Shungin et al. 2015; Matsuyama et al. 2021). Fortunately, some recent applications of Mendelian randomization (a specific type of IV; see Davey Smith and Hemani 2014), propensity score matching (Rosenbaum and Rubin 1983), marginal structural models (Vanderweele et al. 2010), and g-computation (Naimi et al. 2017) seem to mirror a tendency toward more diversity in causal inferential methods in dental research (e.g., Nascimento et al. 2017; Baumeister et al. 2021; Souza et al. 2021; Wu et al. 2021). There is growing use of causal methods in the dental research field, but a wider range of alternative methodological perspectives should be welcomed and encouraged. In particular, as also highlighted by the 2021 Nobel Prize in Economics, alternative methods exist to leverage natural experiments for causal inference from observational data.

Third, while dental research has been promoting the use of directed acyclic graphs (DAGs) to help conceptualize causal thinking (Akinkugbe et al. 2016), comparably little attention seems to have been paid to choosing and applying appropriate data-analytic approaches for causal inference. Make no mistake: there is nothing wrong with promoting and using DAGs to guide causal inference. However, the use of a DAG alone does not in and by itself turn a publication into causal evidence unless supported by a suitable data-analytic approach. Vice versa, the unwary use of complex statistical methods without careful consideration of theoretical pathways to causality is equally misleading. A causal roadmap, a framework for having the question and interpretation clear, includes steps for defining the causal question, assessing its identifiability, estimating statistical parameters, and interpreting them (Petersen et al. 2014; Ahern 2018).

Fourth, similar to other fields of medicine, confusion seems to persist within the dental research community as to the use of causal language (Hernán 2018). To avoid ambiguity and confusion, causal language should be used where appropriate, and it should not be used where inappropriate. Causal and noncausal terms should not be mixed up with each other. The term *causal effect* should explicitly be used when a study has been using robust methods for causal inference. Where causal language is appropriate, editors and reviewers should not urge authors to remove causal wording from publications. Also note that some researchers consider the term *causal association* to be misleading and argue that the word *association* should exclusively be reserved to reporting on noncausal evidence.

If dental research is to secure a robust evidence base for promoting effective oral health interventions, we argue that dental research needs to move beyond its current methodological confinements and expand further toward a much more pluralistic approach to causal inference. Other fields of medicine increasingly exploit the benefits of causal inference from natural experiments, and dentistry cannot lag behind such methodological innovations. This is particularly relevant for complex population-level interventions that are often unamenable for testing via human-made trials.

We call for editors, reviewers, and authors as well as dental academic institutions, dental education associations, the International Association for Dental Research, and other dental research organizations to embrace a much more critically reflective approach to causal inference. Proficiency in causal inference and the appropriate use of causal language should be a core competency for every researcher. Informed by clearly formulated research questions, the full potential of available methods for causal inference should be used (including through observational data), thereby being cognizant of sound theoretical frameworks and pertinent data-analytic approaches.

## Author Contributions

S. Listl, contributed to conception, design, and data interpretation, drafted and critically revised the manuscript; Y. Matsuyama, H. Jürges, contributed to conception, design, and data interpretation, critically revised the manuscript. All authors gave final approval and agree to be accountable for all aspects of the work.

## References

[bibr1-00220345221084283] Ahern J. 2018. Start with the “C-Word,” follow the roadmap for causal inference. Am J Public Health. 108(5):621. 29617622 10.2105/AJPH.2018.304358 PMC5888061

[bibr2-00220345221084283] Akinkugbe AA Sharma S Ohrbach R Slade GD Poole C. 2016. Directed acyclic graphs for oral disease research. J Dent Res. 95(8):853–859. 27000052 10.1177/0022034516639920 PMC4935832

[bibr3-00220345221084283] Angrist J Pischke S. 2008. Mostly harmless econometrics: an empiricists’ companion. Princeton (NJ): Princeton University Press.

[bibr4-00220345221084283] Baumeister SE Freuer D Nolde M Kocher T Baurecht H Khazaei Y Ehmke B Holtfreter B. 2021. Testing the association between tobacco smoking, alcohol consumption, and risk of periodontitis: a Mendelian randomization study. J Clin Periodontol. 48(11):1414–1420. 34472130 10.1111/jcpe.13544

[bibr5-00220345221084283] Cunningham S. 2021. Causal inference: the mixtape [accessed 2021 Dec 12]. https://mixtape.scunning.com/.

[bibr6-00220345221084283] Davey Smith G Hemani G . 2014. Mendelian randomization: genetic anchors for causal inference in epidemiological studies. Hum Mol Genet. 23(R1):R89–R98. 25064373 10.1093/hmg/ddu328 PMC4170722

[bibr7-00220345221084283] Gelman A Imbens G. 2013. Why ask why? Forward causal inference and reverse causal questions. NBER Working Papers No. 19614. National Bureau of Economic Research, Inc. [accessed 2022 January 31]. https://ideas.repec.org/p/nbr/nberwo/19614.html.

[bibr8-00220345221084283] Hernán MA. 2018. The C-word: scientific euphemisms do not improve causal inference from observational data. Am J Public Health. 108(5):616–619. 29565659 10.2105/AJPH.2018.304337 PMC5888052

[bibr9-00220345221084283] Hernán MA Robins JM . 2020. Causal inference: what if. Boca Raton (FL): Chapman & Hall/CRC.

[bibr10-00220345221084283] Huntington-Klein N. 2021. The effect: an introduction to research design and causality [accessed 2021 Dec 12]. https://theeffectbook.net/.

[bibr11-00220345221084283] Listl S Jürges H Watt RG . 2016. Causal inference from observational data. Community Dent Oral Epidemiol. 44(5):409–415. 27111146 10.1111/cdoe.12231

[bibr12-00220345221084283] Matsuyama Y Jürges H Dewey M Listl S. 2021. Causal effect of tooth loss on depression: evidence from a population-wide natural experiment in the USA. Epidemiol Psychiatr Sci. 30:e38. 34030762 10.1017/S2045796021000287 PMC8157508

[bibr13-00220345221084283] Matsuyama Y Jürges H Listl S. 2019. The causal effect of education on tooth loss: evidence from United Kingdom schooling reforms. Am J Epidemiol. 188(1):87–95. 30203091 10.1093/aje/kwy205

[bibr14-00220345221084283] Naimi AI Cole SR Kennedy EH . 2017. An introduction to g methods. Int J Epidemiol. 46(2):756–762. 28039382 10.1093/ije/dyw323 PMC6074945

[bibr15-00220345221084283] Nascimento GG Peres MA Mittinty MN Peres KG Do LG Horta BL Gigante DP Corrêa MB Demarco FF. 2017. Diet-induced overweight and obesity and periodontitis risk: an application of the parametric G-formula in the 1982 Pelotas Birth Cohort. Am J Epidemiol. 185(6):442–451. 28174825 10.1093/aje/kww187

[bibr16-00220345221084283] Petersen ML van der Laan MJ . 2014. Causal models and learning from data: integrating causal modeling and statistical estimation. Epidemiology. 25(3):418–426. 24713881 10.1097/EDE.0000000000000078 PMC4077670

[bibr17-00220345221084283] Raittio E Farmer J. 2021. Methodological gaps in studying the oral-systemic disease connection. J Dent Res. 100(5):445–447. 33646887 10.1177/0022034520982972

[bibr18-00220345221084283] Rosenbaum PR Rubin DB . 1983. The central role of the propensity score in observational studies for causal effects. Biometrika. 70(1):41–55.

[bibr19-00220345221084283] Royal Swedish Academy of Sciences. 2021. Press release about Sveriges Riksbank Prize in Economic Sciences in Memory of Alfred Nobel 2021. October 11, 2021 [accessed 2021 Dec 12]. https://www.nobelprize.org/uploads/2021/10/press-economicsciencesprize2021-2.pdf.

[bibr20-00220345221084283] Seitz MW Listl S Bartols A Schubert I Blaschke K Haux C Van Der Zande MM . 2019. Current knowledge on correlations between highly prevalent dental conditions and chronic diseases: an umbrella review. Prev Chronic Dis. 16:E132. 31560644 10.5888/pcd16.180641 PMC6795069

[bibr21-00220345221084283] Shungin D Cornelis MC Divaris K Holtfreter B Shaffer JR Yu YH Barros SP Beck JD Biffar R Boerwinkle EA , et al. 2015. Using genetics to test the causal relationship of total adiposity and periodontitis: Mendelian randomization analyses in the Gene-Lifestyle Interactions and Dental Endpoints (GLIDE) Consortium. Int J Epidemiol. 44(2):638–650. 26050256 10.1093/ije/dyv075 PMC4817600

[bibr22-00220345221084283] Souza ML Nascimento GG González-Chica DA Peres KG Peres MA. 2021. Counterfactual approach on the effect of metabolic syndrome on tooth loss: a population-based study. J Periodontol [epub ahead of print 13 Aug 2021]. In press. 10.1002/JPER.21-0175 34389993

[bibr23-00220345221084283] Vanderweele TJ Vansteelandt S Robins JM . 2010. Marginal structural models for sufficient cause interactions. Am J Epidemiol. 171(4):506–514. 20067916 10.1093/aje/kwp396 PMC2877448

[bibr24-00220345221084283] Wu SC Ma XX Zhang ZY Lo ECM Wang X Wang B Tai BJ Hu DY Lin HC Wang CX , et al. 2021. Ethnic disparities in dental caries among adolescents in China. J Dent Res. 100(5):496–506. 33283631 10.1177/0022034520976541

